# Bibliometrics of the current state of application of teletechnology in the rehabilitation of patients with heart and large blood vessel disease

**DOI:** 10.3389/fmedt.2024.1382316

**Published:** 2025-01-22

**Authors:** Sican Wang, Ping Yu, Xuemei Zhou, Yuan Yuan, Jian Chen, Dongmei Chen, Jingyan Liang, Li Xu

**Affiliations:** ^1^ School of Nursing & School of Public Health, Yangzhou University, Yangzhou, China; ^2^ Department of Nursing, Yangzhou First People’s Hospital, Affiliated Hospital of Yangzhou University, Yangzhou, China; ^3^ Surgery Department, Yangzhou First People’s Hospital, Affiliated Hospital of Yangzhou University, Yangzhou, China; ^4^ Cardiac and Large Vascular Surgery, Yangzhou First People’s Hospital, Affiliated Hospital of Yangzhou University, Yangzhou, China; ^5^ Institute of Translational Medicine, Medical College, Yangzhou University, Yangzhou, China; ^6^ Jiangsu Key Laboratory of Integrated Traditional Chinese and Western Medicine for Prevention and Treatment of Senile Diseases, Yangzhou University, Yangzhou, China; ^7^ Clinical Trial Institution, Nanjing Drum Tower Hospital, The Affiliated Hospital of Nanjing University Medical School, Nanjing, Jiangsu, China

**Keywords:** teletechnology, telemetry, cardiac surgery, heart disease, cardiac rehabilitation, rehabilitation, bibliometrics

## Abstract

The first publication on the use of teletechnology in heart and large blood vessels dates back to 1961. Since then, the study of teletechnology in heart and large blood vessels has become popular, and the number of publications has drastically increased. Hence, it is imperative to establish a comprehensive research framework that enables researchers and other stakeholders to understand the use of remote technologies in heart and large blood vessels. To bridge this gap, bibliometrics was used, a novel approach to determine the most prolific countries, institutions, journals, authors, source topics, funding agencies, and the most popular category of remote technologies and solutions for disease rehabilitation. The corpus was extracted from the WOS core database and analyzed using CiteSpace 6.2R7 and VOS Viewer 1.6.18 versions. The number of publications has grown since the start of the 21st century, with the United States, the United Kingdom, and Italy being the most productive nations. The most commonly used remote technology was a 24 h dynamic electrocardiogram (ECG) and ambulatory blood pressure monitoring. The most researched objective indicators were heart rate, blood pressure, and cardiac output. The primary research focused on daily life, physical activity, exercise endurance, and quality of life. Moreover, heart failure and coronary artery disease were the most extensively researched diseases.

## 1 Introduction

Cardiovascular disease (CVD) is a broad term for diseases involving the heart and the cardiovascular system (1). CVDs include coronary artery disease, heart failure, cardiomyopathy, arrhythmia, aortic aneurysm, peripheral artery disease, and venous thrombosis (1, 2).

The age-standardized prevalence of CVD in South Asia increased from 5,881.0 per 100,000 people in 2022 to 11,342.6 per 100,000 people in Central Asia. The age-standardized CVD mortality rate per 100,000 people varied from 73.6 in high-income nations in the Asia-Pacific area to 432.3 in Eastern Europe. Between 1990 and 2022, there was a significant reduction (34.9%) in the worldwide death rate attributed to CVD (3). Outside of Africa, CVD is the leading cause of death worldwide (1). In the United States, CVD affects 26% of adults aged 20–39, 53% of those aged 40–59, 77% of those aged 60–79, and over 85% of those aged 80 and older, among individuals with hypertension. In adults without hypertension, CVD prevalence rises from 1.5% in the 20–39 age group to 36% in those aged 80 and above. The highest prevalence of CVD is observed in adults over 80 years of age (4).

Cardiovascular surgery is a crucial therapy for CVD, including the heart and large blood vessels. The postoperative rehabilitation process is of great significance since it is a long-term and ongoing procedure for patients recovering from the procedure. The application of telemetry electrocardiogram (ECG) monitoring and telerehabilitation in the postoperative rehabilitation of patients with heart and large blood vessel disease is crucial.

The first documented literature on the utilization of teletechnology in patients with CVD dates back to 1961. During this time, an article established the concept of telemetry ECG, a technology that enables the transmission of a patient's ECG to a recording device through air (5). Conversely, due to the limitations of regional medical resources and the patient's condition, in-hospital rehabilitation cannot meet the rehabilitation needs of patients. Furthermore, the rapid progress of healthcare technology, along with the accessibility of novel therapies utilizing remote technology, has led to significant growth in the implementation and study of teletechnology in the treatment of heart and large blood vessel diseases and related fields.

Traditional reviews focus on the article's content without delving into the topic issues and partnerships. The bibliometric analysis employs software to extract various data from the literature, including authors, countries, institutions, keywords, cited authors, cited journals, etc. This data was then used to generate visual representations depicting the relationship between knowledge development and structure. Bibliometric analysis uses visualization technology to explore, analyze, construct, describe, and present knowledge and its interconnections. Furthermore, it is a statistical technique that condenses data and information, providing a concise and comprehensive overview of the overall trends in a specific subject through quantitative analysis (6). This method effectively investigates the progression of a discipline (7). The currently employed bibliometric analysis software, including CiteSpace (8) (Drexel University in Philadelphia, USA) and VOSviewer (9) (Leiden University in the Netherlands), can identify potential collaborators via software-generated collaboration networks and co-occurrence networks. The burst term provides scholars with a dynamic development process in the field. This progressive advancement enables academics to thoroughly understand the present status of research on this topic (10) and predict future research focus areas. The bibliometric review investigated remote technology's current status and future heart and large vessel disease trends since the 21st century. This technique allowed the analysis and evaluation of important research articles and gave valuable insights for future studies.

## 2 Materials and methods

### 2.1 Search strategy

Due to the extensive period of relevant articles, to ensure the quality and timeliness of the articles cited in the study, the publication time of the analyzed literature was limited to after the year 2000. Web of Science Core Collection was the source of bibliographic data; researchers widely accept this source as a high-quality digital literature database and is generally regarded as the most appropriate database for bibliometric analysis. Researchers conducted additional searches in databases such as PubMed and Scopus to remove duplicates but found no new articles. Ultimately, only the search results from the WOS database were used. The selected citation indexes were Science Citation Index Expanded (SCI-EXPANDED) and Social Sciences Citation Index (SSCI) to ensure that the retrieved data was comprehensive and accurate. Search strategy: TS = (Cardiac surgery or (surgery, cardiac or cardiovascular or heart disease or cardiopulmonary or heart-lung or Cardi* or congenital heart disease or heart valve surgery or Cardiac valvular surgery or Heart Valve Surgery or Cardiac valve replacement or valvular replacement surgery or Surgery for congenital heart disease or Coronary Artery Bypass Grafting or CABG or Coronary artery bypass grafting or OPCAB or off-pump coronary artery bypass grafting or peripheral artery or peripheral arterial)) AND TS = (Telemetry or (Telemetry technology or wireless ECG monitoring or Remote Monitoring or telemonitoring or ambulatory monitoring)) AND TS = (cardiac rehabilitation or (early rehabilitation or early recovery or early getting out of bed or rehabilitation or exercise)). Articles, including experimental studies such as RCTs and observational studies, as well as reviews,were selected as the document types. After the removal of deduplicates, 1,002 articles were finally obtained.

### 2.2 Analytical tools

The analysis tool in Microsoft Excel 2019 was utilized to generate charts depicting the cumulative yearly publications from 2000 to 2023. These charts were used to examine the worldwide production and trends in telemetry linked to heart and large blood vessel disease, specifically focusing on papers on patient recovery. CiteSpace 6.2R7 and VOS Viewer 1.6.18 were used to prepare network diagrams, extract, and analyze the number of publications (including outputs, countries, institutions, journals, and authors), frequency of citation (including co-cited authors and burst term), as well as track research trends and hot spots. Both these software were for data visualization based on the Java platform. CiteSpace was used to generate collaborative networks (including countries, institutions, and authors), co-cited networks (co-cited authors), co-occurrence networks (strongest burst citations and keywords), and dual-map overlap. The VOSviewer tool produces a visual representation of clusters. In this representation, the color of each node indicates the cluster it belongs to, the size of each node indicates the frequency of co-occurrence, and the lines connecting the nodes show the co-occurrence or co-citation relationship. The thicker or thinner lines between the nodes represent the intensity of co-occurrence (or co-citation).

### 2.3 Research ethics

In this review, bibliometric analysis was performed. All data sources were available online and did not involve animal or human subjects. Therefore, permission from the Ethics Committee was not required.

## 3 Results and discussion

### 3.1 Spatial distribution of research

#### 3.1.1 Analysis of publications outputs and citations

**Figure 1:**
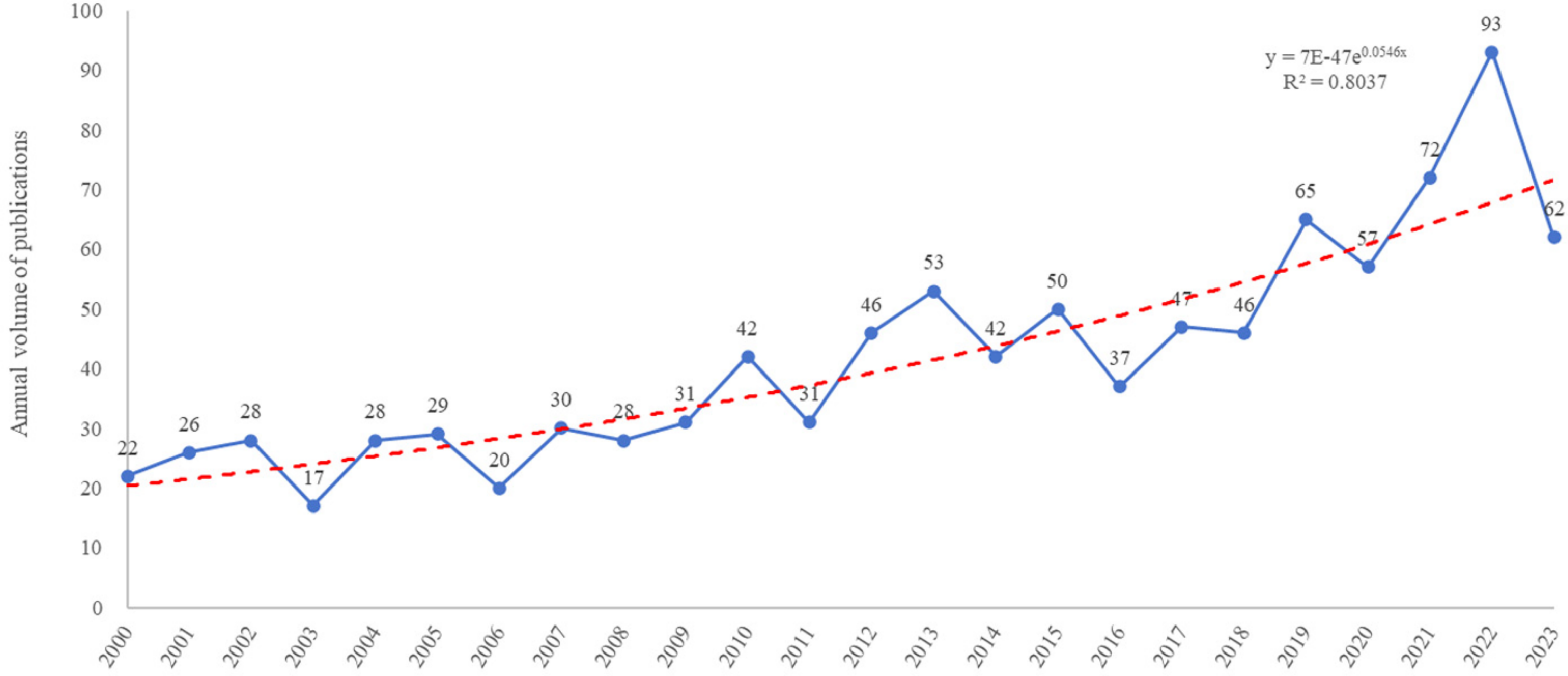
Annual cumulative publications (2000–2023).

The Web of Science Core Collection included 1,002 publications that met the inclusion criteria between January 1, 2000 and December 31, 2023. These included 890 articles and 112 reviews, with an average annual output of 42 articles. Microsoft Excel was used, and graphs of the number of publications were prepared (Figure 1). The number of articles published and cited increased consistently, with an average yearly growth rate of 281.8% (from 22 in 2000 to 62 in 2023).

#### 3.1.2 Analysis of countries, institutions, and research cooperation

**Figure 2:**
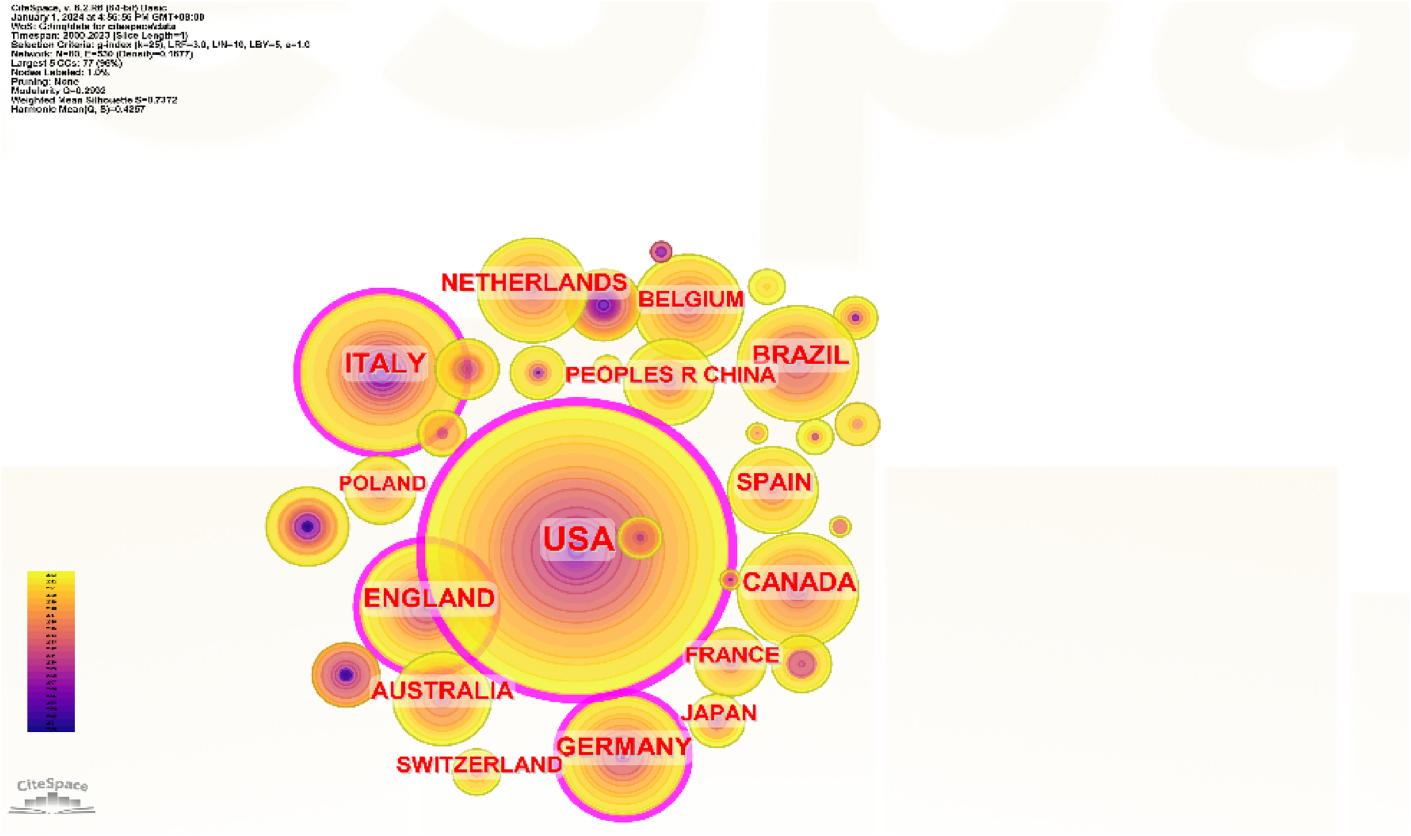
Collaborative networks of countries.

**Figure 3:**
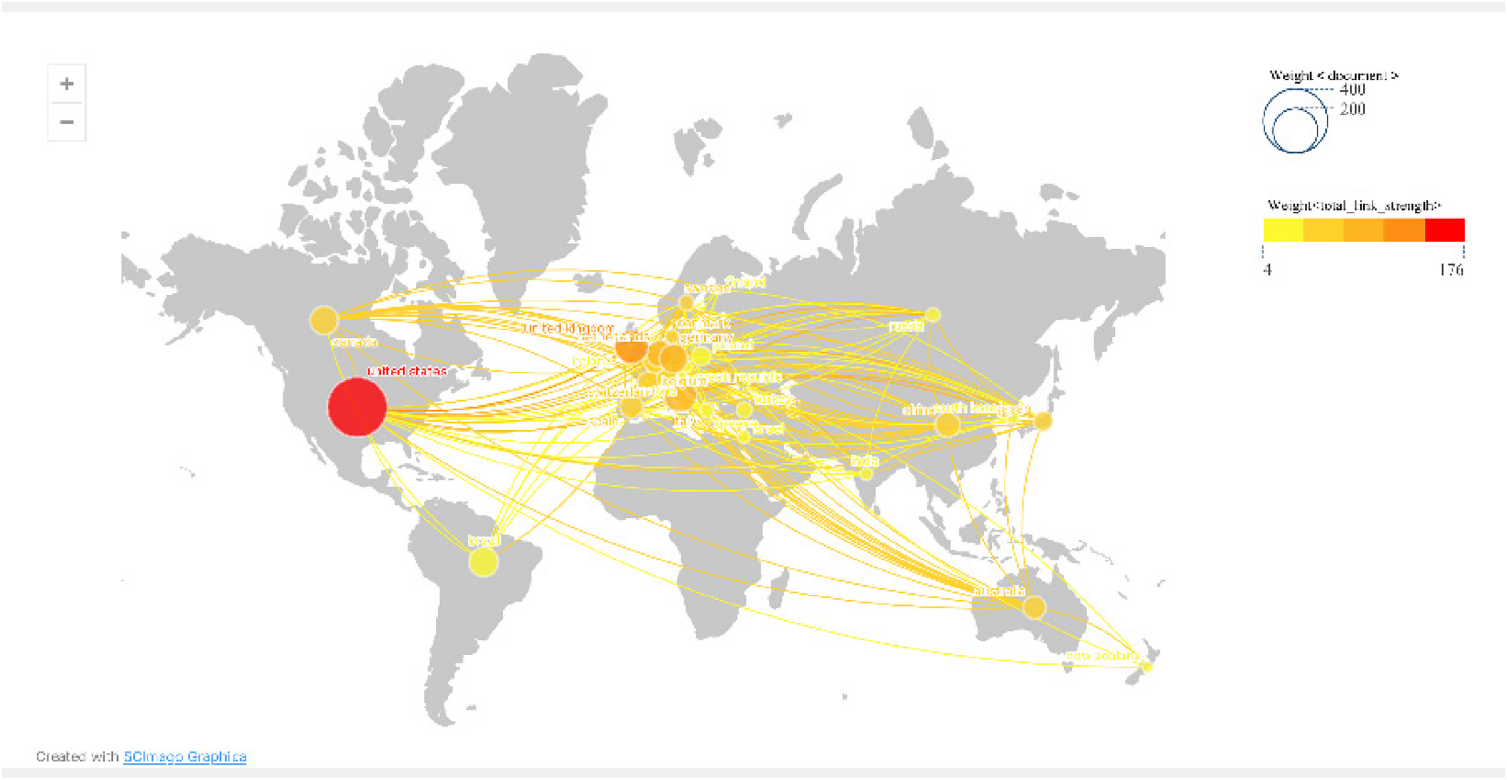
Scimago Graphica national visualization map.

In Figure 2, the size of the citation tree rings represents the number of published papers, the color represents the publication time, and the lighter the color, the closer the publication time. The purple circle represents betweenness centrality (betweenness centrality > 0.1); betweenness centrality is one of the critical concepts in bibliometric research, and having a high degree of betweenness centrality is usually the key to linking two countries, institutions, or fields. It is also known as a turning point in Citespace (11). Figure 3 is the visualization map of National Geographic based on Scimago Graphica. The size of the circle corresponds to the number of published papers. The closer the color to red, the higher the number of published papers.

An examination of the national collaborative networks (Figures 2, 3) utilizing CiteSpace demonstrated that 77 countries employed teletechnology and contributed to publications on the rehabilitation of patients with heart and large blood vessel disease. The leading five countries, in terms of publication count, were the United States, the United Kingdom, Italy, Brazil, and Germany. Brazil is a developing country in this list, and the remaining four countries are developed. Thus, one can infer that developed countries play a more crucial role in this field of research. The United States, Germany, Italy, the United Kingdom, France, and Malaysia are among the top five nations in terms of centrality and significantly impact worldwide research collaboration. The fact that the five leading countries in publication output are predominantly developed is noteworthy. However, further exploration of the implications of this concentration could be valuable, particularly to understand whether active efforts are being made to engage a greater number of developing countries in research initiatives.

**Figure 4:**
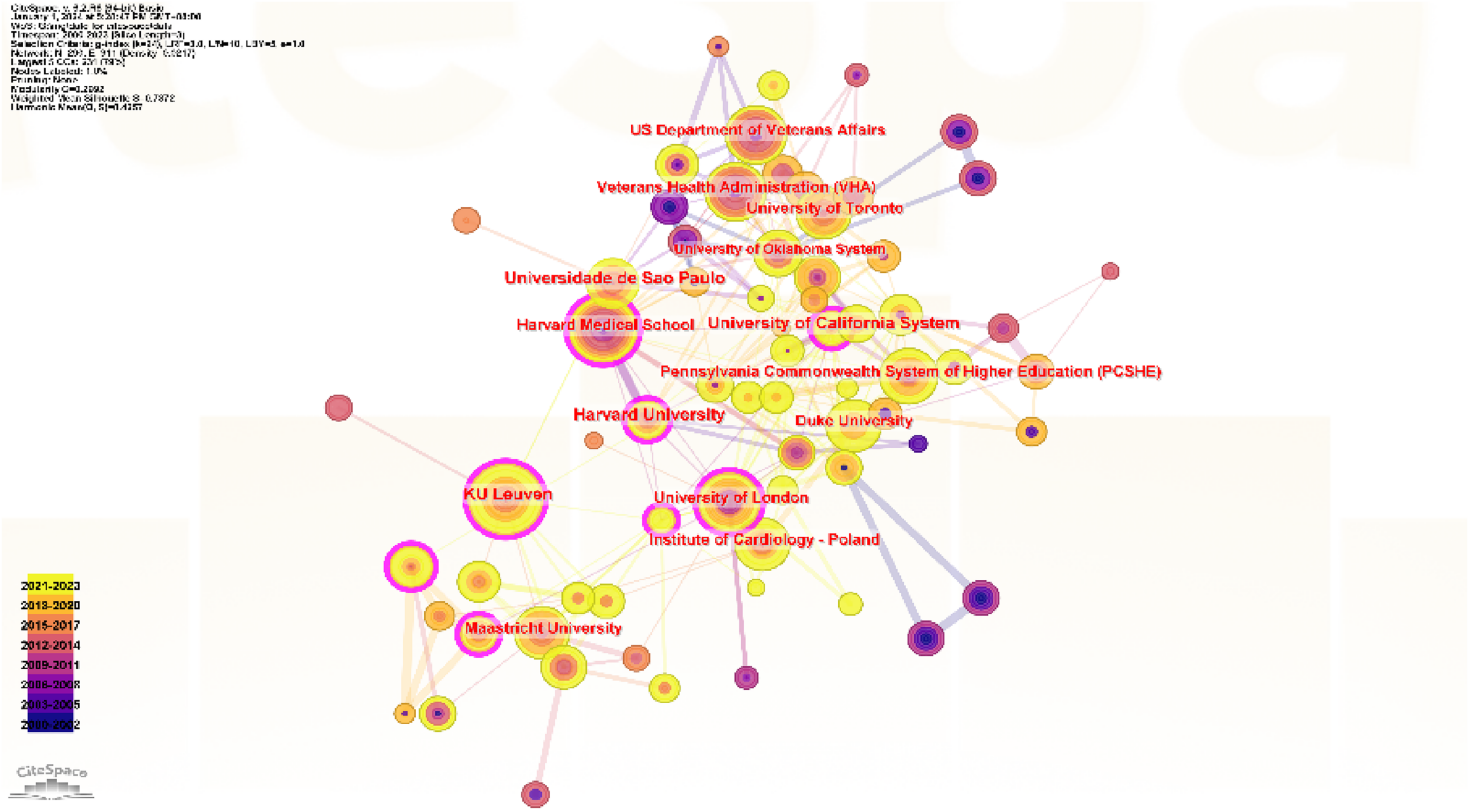
Collaborative networks of institutions.

Figures 3, 4 and Table 1 present a map of institutional collaboration networks for each country and region. The United States dominated the list of universities with the largest number of published papers, with Harvard University leading the way with 27 publications. Other notable institutions were Universidade de Sao Paulo, with 25 papers; KU Leuven, with 22 papers; Harvard Medical School and the University of California System, both with 19 papers. The CiteSpace was used to calculate the betweenness centrality of research institutions. It was found that three of the top five institutions for centrality were from Europe (Table 1), and the Assistance Publique Hopitaux Paris (APHP) ranked first with a mediation centrality of 0.12.

**Table 1:** Top 10 publishing institutions with betweenness centrality.

Rank	Institution	Country	Centrality
1	Assistance Publique Hopitaux Paris (APHP)	France	0.12
2	CIBER—Centro de Investigacion Biomedica en Red	Spain	0.12
3	Harvard University	USA	0.11
4	KU Leuven	Belgium	0.08
5	University of California System	USA	0.08
6	University of London	UK	0.08
7	Columbia University	USA	0.06
8	Duke University	USA	0.05
9	US Department of Veterans Affairs	USA	0.05
10	Universidade de Sao Paulo	Brazil	0.04

APHP, a university hospital trust operating in and around Paris, is the largest hospital system in Europe and one of the largest in the world (12). This hospital receives an average of more than 10 million patients annually and employs more than 90,000 individuals in 44 hospitals (13). Thus, more than 10 million patient visits are received annually (14). APHP is connected to the University of Paris with its seven faculties of medicine, two faculties of dentistry, and two faculties of pharmacy. Additionally, it serves as the primary medical facility for the capital city and its surrounding areas. The Chinese Biomedical Research Center has the second position, followed by Harvard University, KU Leuven, and the University of California system, which occupy the third, fourth, and fifth positions, respectively. The centrality of the top three institutions was greater than 0.1, indicating good cooperation among institutions. The KU Leuven and the University of California system was less than 0.1, suggesting the future need for enhanced inter-agency cooperation.

#### 3.1.3 Prolific source titles

A total of 449 journals have published papers on the significance of teletechnology in rehabilitating patients with heart and large blood vessel disease. Table 2 lists the top 10 academic journals that have published papers on the study of teletechnology in rehabilitating patients with heart and large blood vessel disease. The International Journal of Cardiology ranked first in terms of publishing, with a total of 17 articles. The Journal of the American College of Cardiology significantly contributed to the field due to its high citation rate and impact factor (If = 24). The average impact factor of the top 10 journals was 6.18. The majority of the journals were from the second quarter and beyond, while most of the others were from the first quarter. Most journals were in the United States, and the top 10 journals were in developed nations. Seven of the top ten journals published articles in the first quartile, with the remaining three journals in the second quartile, indicating that the quality of the articles included in this study is relatively high.

**Table 2:** Titles of the most prolific sources.

Source Title	Number of Publications	Impact Factors (SJR—Scopus 2023)	H-Index	Quarter
International Journal of Cardiology	17	3.2	108	Q1
Journal of the American College of Cardiology	15	24.2	394	Q1
American Journal of Hypertension	14	2.8	127	Q1
European Journal of Preventive Cardiology	13	7.1	88	Q1
Frontiers in Physiology	13	4	75	Q2
Archives of Physical Medicine and Rehabilitation	13	3.3	169	Q1
American Journal of Cardiology	12	2.3	206	Q2
Sensors	12	3.4	132	Q1
Blood Pressure Monitoring	12	1.2	57	Q2
Journal of Hypertension	12	3.1	160	Q1
Europace	12	7.2	92	Q1

**Figure 5:**
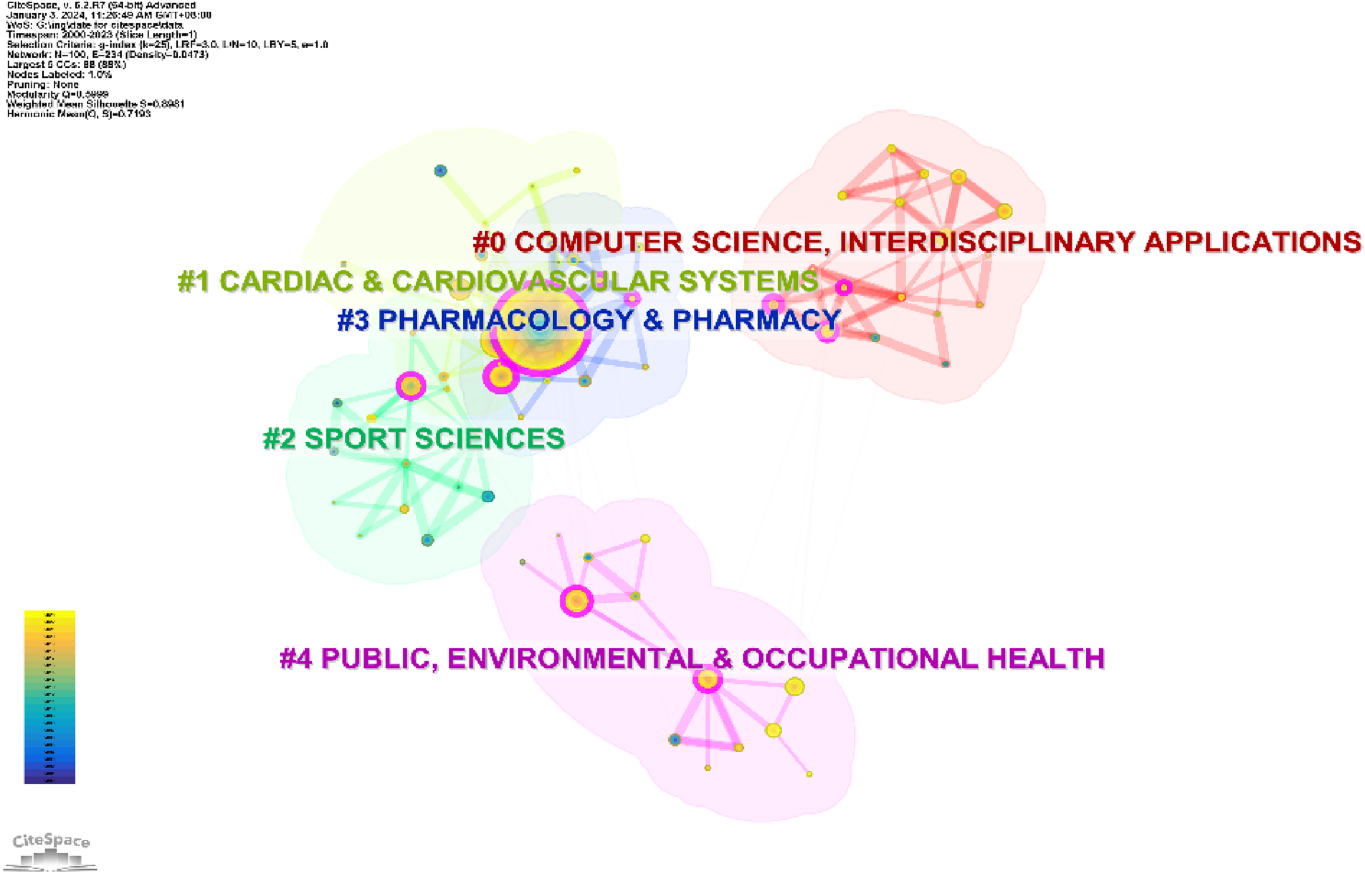
Cluster map of citing literature.

**Figure 6:**
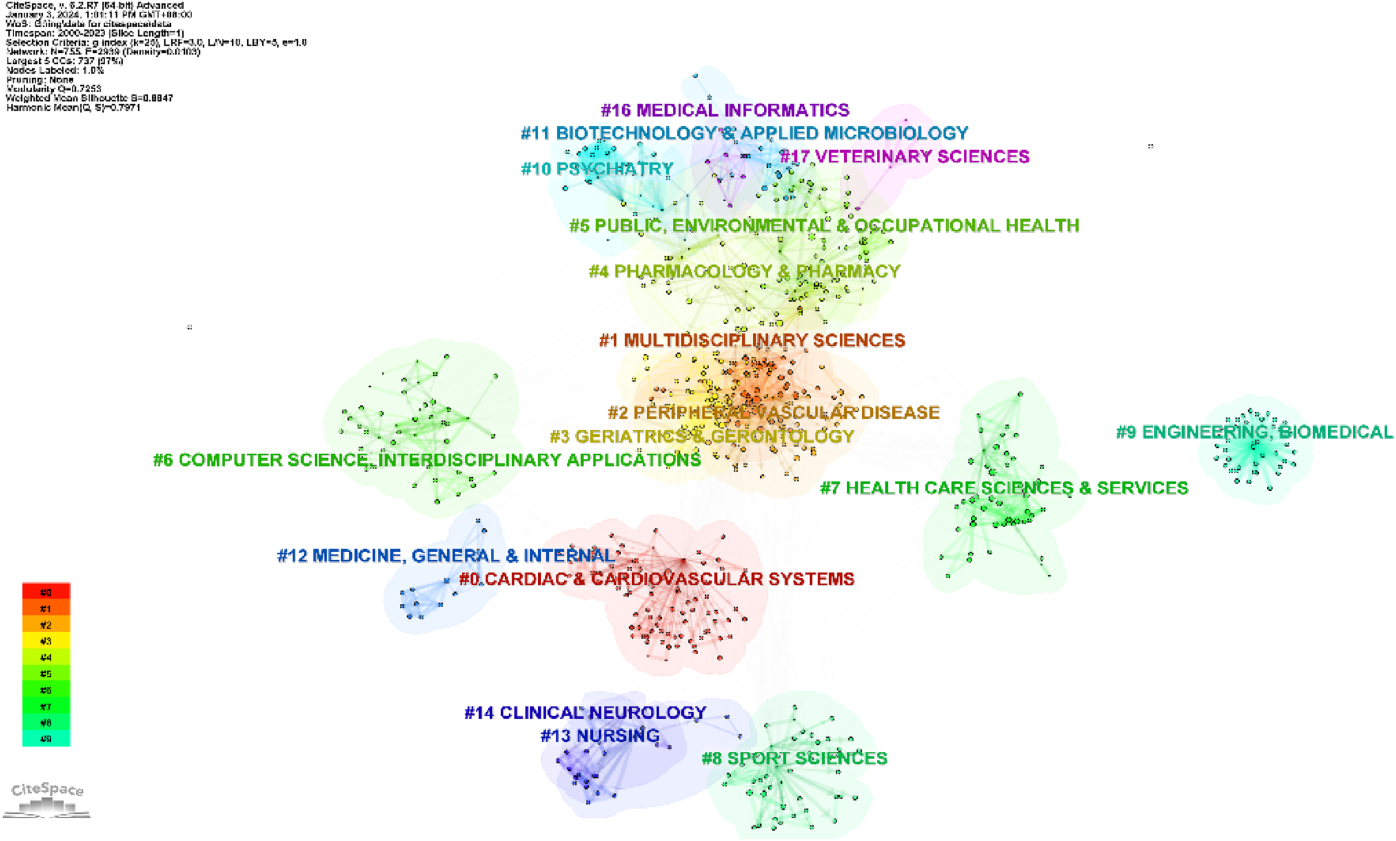
Cluster map of cited articles.

The 1,002 cited publications were classified into five clusters (Figure 5): “Computer Science and Interdisciplinary Applications”, “Cardiac and Cardiovascular System”, “Sports Science”, “Pharmacy and Pharmacology”, and “Public Environment and Occupational Health”. The cited articles (33,532) were grouped into 17 key clusters, with the order of the first five being Cardiac and Cardiovascular System, Multidisciplinary Sciences, Peripheral Vascular Diseases, Geriatrics and Gerontology, and Pharmacy and Pharmacology (Figure 6).

**Figure 7:**
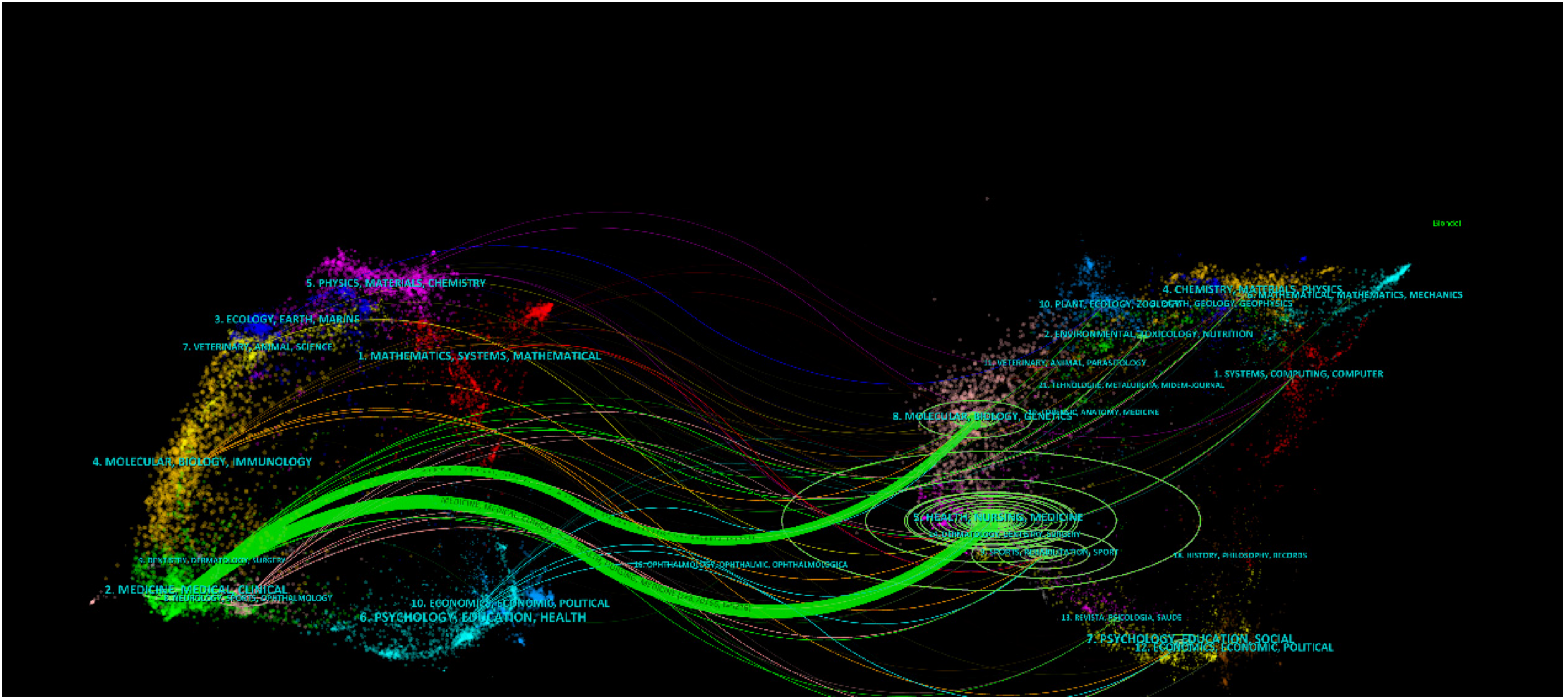
Dual-map overlap of journals.

Figure 7 displays a double mapping of journals. The left side presents the citation map, the right side shows the cited map, and the curve indicates the citation link. Link trajectories offer a comprehensive picture of the field from several disciplines, and the *z*-score feature displays smore fluid trajectories. The score increases as the link becomes closer. In this particular context (11), publications in the domains of medicine, medical, and clinical (Green Tracks) were impacted by publications in the domains of molecular biology and genetics (*z* = 2.09, *f* = 9,555), as well as health, nursing, and medicine (*z* = 8.70, *f* = 35,512).

**Figure 8:**
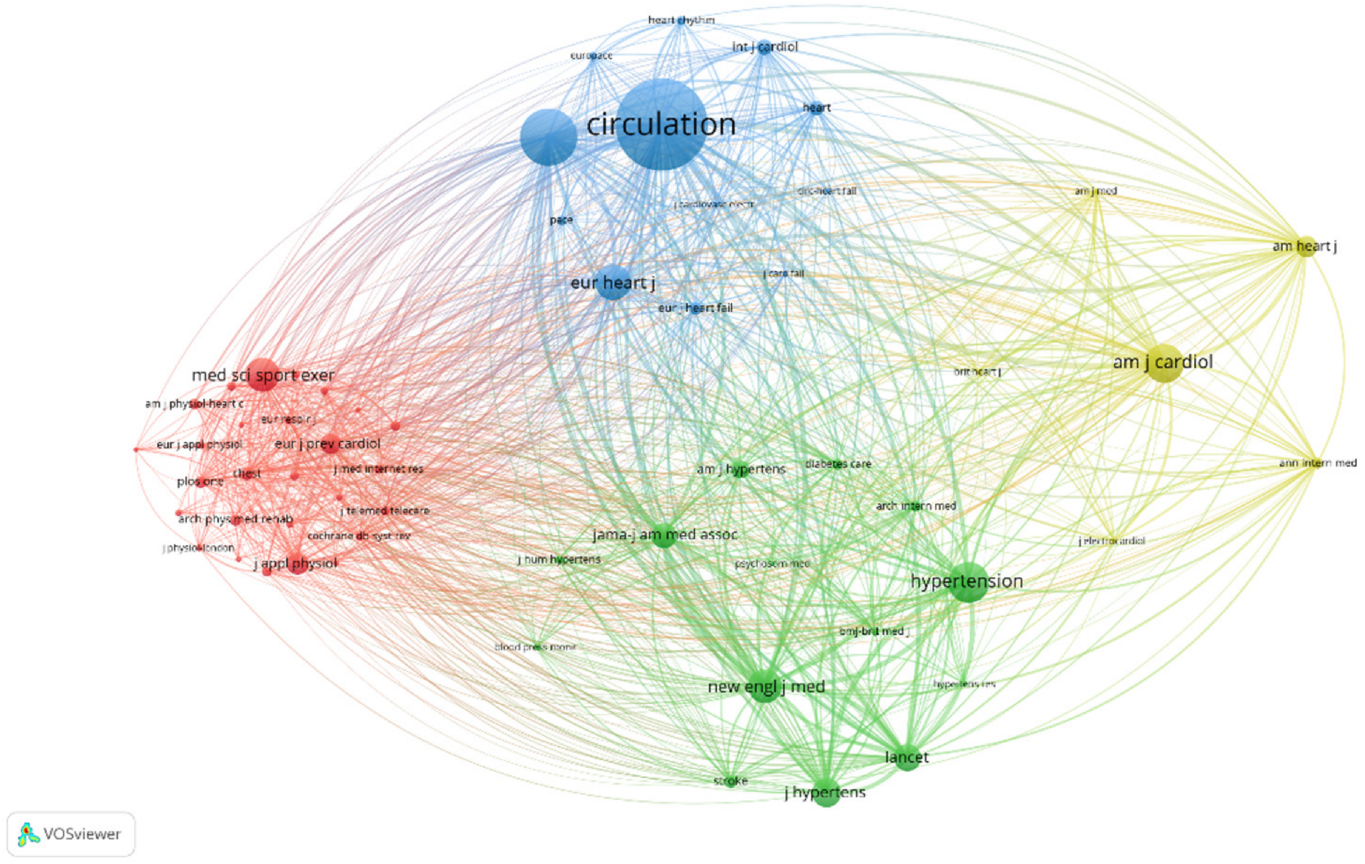
Cluster map of co-cited journals.

To further analyze the journal co-citation, the VOSviewer software was used for a co-citation analysis of 59 journals with a threshold of 100 times. The results were presented in Figure 8, where four clusters correspond to the four colors in the figure. The Blue Group primarily served as a journal in the domain of heart and CVD, with a specific focus on the utilization of therapeutic methods for cardiac and cardiovascular conditions, rehabilitation technologies, and integrated care tools for disorders affecting the heart and large blood vessels. The red group indicated mainly a journal in kinesiology and general studies, with more research in the rehabilitation of cardiac and cardiovascular-related diseases, assessment of exercise, and quality of life. Moreover, the green cluster comprised a comprehensive journal that focused on peripheral vascular and internal medicine, as well as clinical medicine, offering several important clinical and laboratory trials to provide information on strategies to enhance patient outcomes. According to Figure 8, the most cited was CIRCULATION (2,354 times), followed by Journal of the American College of Cardiology (1,409 times), and American Journal of Cardiology (945 times).

#### 3.1.4 Prolific source and co-cited author

**Figure 9:**
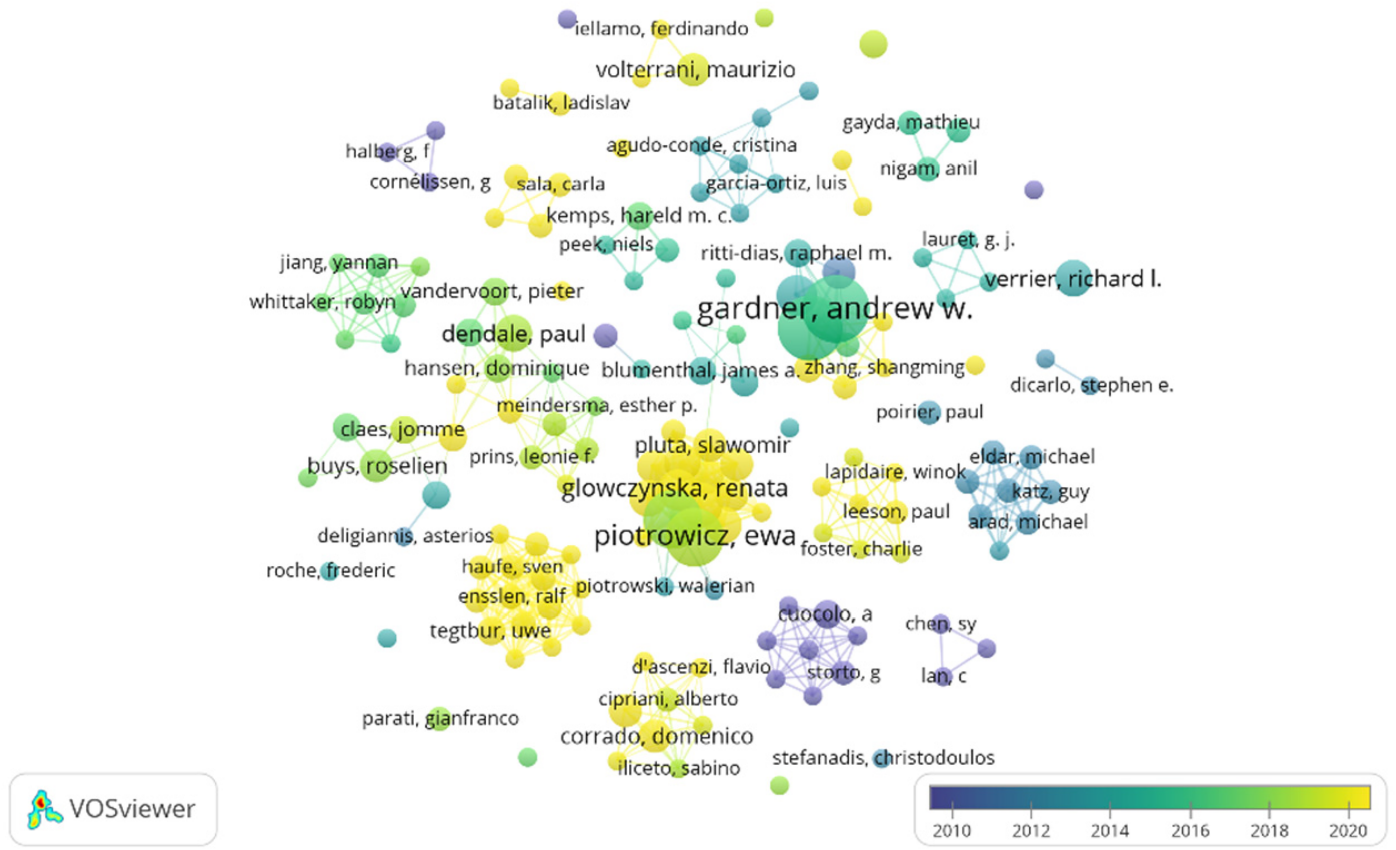
The number and year of authors’ publications.

Author-collaboration mapping was generated employing CiteSpace (Figure 9), and it was found that 6,127 authors were involved in 1,002 papers. The node size represented the number of publications, and the color indicated the year of publication. The article's publication date is indicated by the proximity of the color to blue, with a closer color indicating an earlier publication. Conversely, the author's publication history is shown by the distance of the color to yellow, with a closer color indicating a more recent publication. Table 1 lists the top five authors by volume, including Gardner Andrew. W, ranking first (16 papers), Montgomery, Polly. S. ranking second (15 papers), and Piotrowicz, Ewa ranking third (13 papers). Gardner, Andrew W., Montgomery, and Polly S were the first and second most cited authors, with the citations being 645 and 628, respectively, and Parker, Donald E. had 539 citations, among the top five cited authors (Table 3). Among the co-cited authors, the first rank was held by Gardner, AW, from the University of Oklahoma Health Sciences Center, with 645 citations, and the second rank was held by Montgomery, Polly, from Penn State College of Medicine, with 628 citations. Toudor-Locke C. had the highest centrality of 0.29 among all co-cited authors. Following closely were Koehler F. with a centrality of 0.21, Fletcher G. F. with 0.2, Schultz M. G. with 0.18, and Piotrowicz E. with 0.12. These scholars were very prominent in their respective domains, as seen in Table 3.

**Table 3:** Top 5 cited authors and co-cited authors.

Pank	Author	Count	Co-cited Author	Cite Times	Co-cited Author	Centrality
1	Gardner, Andrew W.	16	Aardner, Andrew W.	645	TUDOR-LOCKE C	0.29
2	Montgomery, Polly S.	15	Montgomery, Polly S.	628	KOEHLER F	0.21
3	Piotrowicz, Ewa	13	Parker, Donald E.	539	FLETCHER GF	0.2
4	Piotrowicz, Ryszard	13	Blevins, Steve M.	492	SCHULTZ MG	0.18
5	Opolski, Grzegorz	9	Blumenthal, James A.	426	PIOTROWICZ E	0.12

#### 3.1.5 Most prolific funding bodies

Since WoS only started to add funding confirmation information in 2008, the present analysis of funding agencies was from 2008 to 2023. Among the 200 funded institutions, the largest output of research was produced by the United States Department of Health and Human Services (*n* = 118), the National Institutes of Health (NIH) of the United States (*n* = 115), the National Institute of Heart, Lung, and Blood (United States; *n* = 49), the National Institute on Aging (United States; *n* = 31), the National Council for the Development of Science and Technology (CNPQ; Brazil; *n* = 29), the Foundation for Human Resource Development in Higher Education (Brazil; *n* = 22), the São Paulo Research Foundation (Brazil; *n* = 20), the National Research Resource Center of the United States National Institutes of Health (*n* = 19), the European Union (*n* = 18), FWO Belgium (*n* = 13), the National Natural Science Foundation of China (*n* = 12), the Canada Institutes of Health Research (*n* = 11), the United States National Institutes of Health NIHR (*n* = 11), the United Kingdom Research Innovation UKRI (*n* = 11), and the United States Heart Association (*n* = 10). Notably, the 10 most productive funding agencies were 50% from the United States, 30% from Brazil, 20% from Europe, and no funding agencies from Asia.

A total of 802 studies were included from 2008 to 2023, of which 545 were funded, accounting for 67.96%. The proportion of funding was found to be significantly higher than that of other areas (15).

### 3.2 Content analysis

#### 3.2.1 Inductive content analysis: most prolific research category and themes

**Figure 10:**
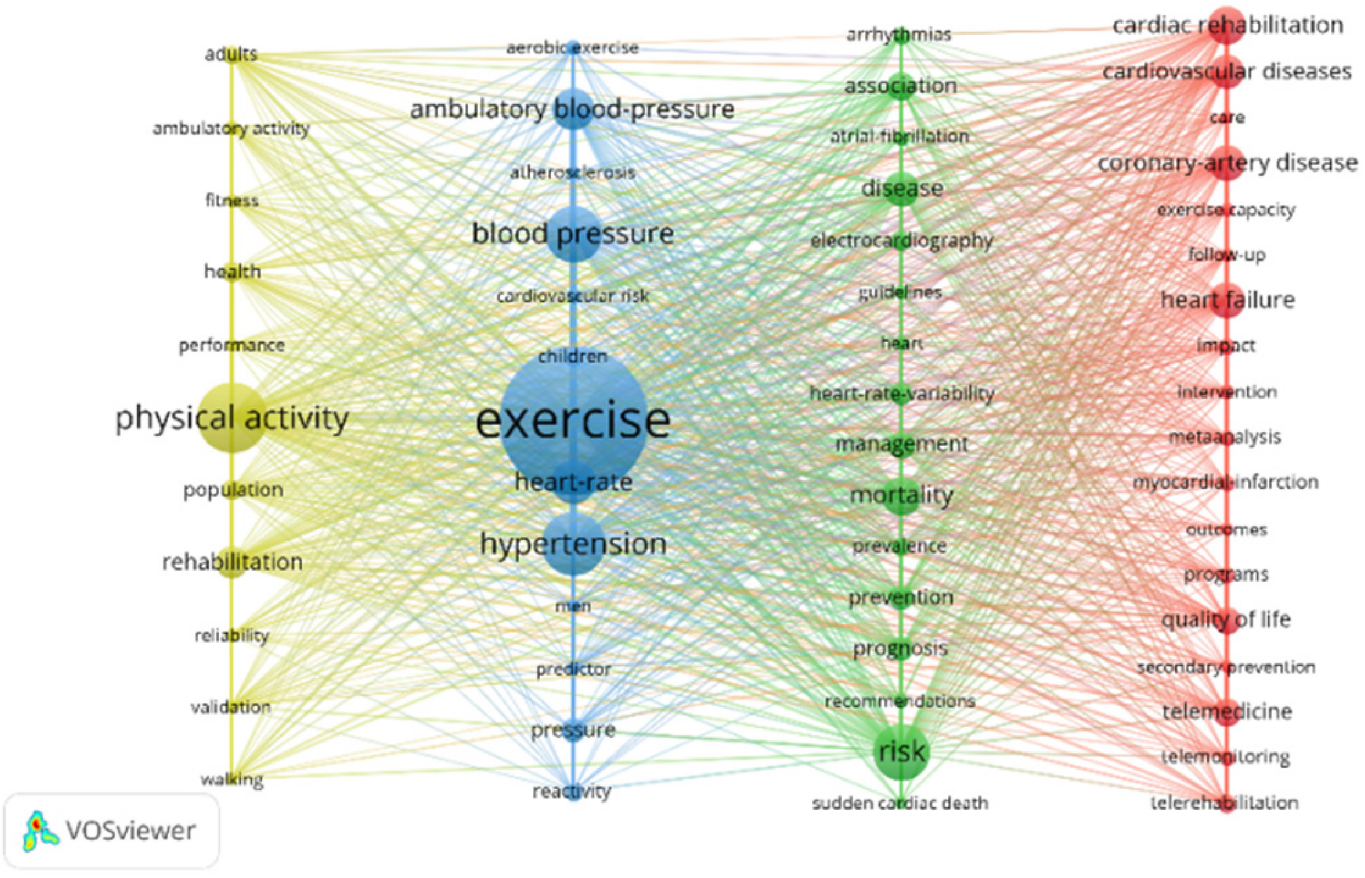
Authors’ keyword map.

Through content analysis, the four themes were obtained (Figure 10 and Table 4).

**Table 4:** Most prolific research category and themes.

Color	Representative Author Keywords (Codes)	Categories	Themes
Yellow (*n* = 11)	Physical activity (165), rehabilitation (76), health (50), population (49), adults (44), ambulatory activity (35), performance (34), fitness (32), reliability (32), validation (31), walking (30)	24 h under continuous dynamic ECG monitoring evaluate the safety of the patients with congenital heart adult sports activities; Home-based exercise based on walking with remote electrocardiogram monitoring; A wearable cardioverter-defibrillator remotely tracks patient physical activity; Sedentary behavior and 24 h ambulatory blood pressure monitoring; Outdoor exercise real-time physiological monitoring after PCI; Association of associated increases in physical activity with cardiorespiratory fitness and heart rate recovery;	Exercise degree and activity safety were evaluated by remote monitoring.
Blue (*n* = 13)	Exercise (346), hypertension (149), blood pressure (134), heart rate (104), ambulatory blood pressure (100), pressure (57), children (41), reactivity (41), aerobic exercise (35), cardiovascular risk (32), men (31), predictor (31), atherosclerosis (30)	Electrogram telemetry was used to compare the effects of exercise in adolescents with heart disease. Heart rate monitoring exercise at home; Under the 24 h ambulatory blood pressure monitoring in patients with sports capacity and the relationship of blood pressure; Effects of exercise training on nocturnal blood pressure decline; Aerobic exercise lower levels of 24 h ambulatory blood pressure patients with high blood pressure;	Ambulatory blood pressure monitoring was used to evaluate the relationship between exercise capacity and blood pressure during exercise.
Green (*n* = 16)	Risk (132), mortality (93), disease (84), association (67), management (65), prevention (59), prognosis (58), heart-rate-variability (53), electrocardiography (49), prevalence (44), arrhythmias (43), atrial-fibrillation (38), recommendations (35), guidelines (32), heart (32), sudden cardiac death (31)	Wearable devices under the electrocardiogram monitoring of postoperative patients with weak heart rate response parameters; 48 h of dynamic electrocardiogram monitoring myocardial ischemia; Duration of heart postoperative convalescence telemetry monitoring atrial fibrillation; To investigate the effect of exercise rehabilitation on the risk of sudden cardiac death under 24 h dynamic electrocardiogram monitoring. Based on a wireless sensor network (WSN) body electrocardiogram on cardiovascular disease prevention and early diagnosis; the intensity of daily peak energy expenditure estimated by 24 h Holter; The long-term dynamic electrocardiogram for atrial fibrillation frequency tracking; Patients with high risk of secondary prevention were identified by 24 h dynamic electrocardiogram.	Dynamic electrocardiogram monitoring and evaluation-related risk factors.
Red (*n* = 18)	Cardiac rehabilitation (89), heart failure (86), coronary-artery disease(85), cardiovascular diseases (80), telemedicine (67), quality of life (65), telerehabilitation (44), programs (40), impact (39), meta-analysis (37), myocardial infarction (37), telemonitoring (37), intervention (35), exercise capacity (34), follow-up (34), care (31), secondary prevention (31), outcomes (30)	Telemedicine instead of outpatient service The virtual consultation and remote monitoring; Home-based cardiac rehabilitation under remote monitoring; Patients did aerobic exercise under Holter monitoring after coronary artery bypass grafting. Domestic cardiac rehabilitation patients’ quality of life increased using wearable devices; Wearable monitoring equipment under family cardiac rehabilitation in patients with follow-up; In the secondary prevention program for remote monitoring of patients with cardiovascular disease in the guidance and supervision; Compliance of CR patients based on eHealth; The self-care behavior of patients was improved under telemedicine intervention; Remote monitor and control the movement of the heart disease patients with physical ability and quality of life measure results;	Remote technical application in home cardiac rehabilitation effect and patient compliance.

Exercise degree and activity safety were evaluated by remote monitoring:

Including 24-h continuous Holter monitoring to assess the safety of physical activity in adults with congenital heart disease. This pertains to the correlation between heightened physical activity, cardiovascular well-being, and the connection between heart rate recovery.

The investigation used ambulatory blood pressure monitoring to assess the correlation between exercise capacity and blood pressure levels during physical activity:

The literature on blue clusters primarily focused on studying the effects of exercise in adolescents with heart disease. This included comparing exercise with electrocardiogram telemetry, monitoring heart rate during home exercise, and evaluating the relationship between exercise capacity and blood pressure using 24-h ambulatory blood pressure monitoring. This allowed the assessment of the impact of exercise training on the decline of blood pressure at night and examined the effect of aerobic exercise on reducing 24 h ambulatory blood pressure in hypertensive patients. Commonly used methods to assess cardiopulmonary function and physical activity levels before recovery included a 6 min walk test and a cardiopulmonary function test. The articles also included studies on the effects of different training modalities, like endurance training, dynamic resistance training, combined endurance and resistance training, and isometric resistance training, on adult resting blood pressure.

Dynamic electrocardiogram monitoring and evaluation-related risk factors:

The green clustering of records involves using wearable devices to monitor the ECG of postoperative patients with poor heart rate response (16). The purpose was to monitor myocardial ischemia and cardiac postoperative convalescence using a 48-h dynamic electrocardiogram. Additionally, this continuously monitors atrial fibrillation using telemetry and tracks the frequency of atrial fibrillation using long-term dynamic electrocardiogram. Furthermore, a 24 h dynamic electrocardiogram was used to identify high-risk secondary prevention patients.

Remote technical application in home cardiac rehabilitation effect and patient compliance:

The literature in the red cluster mostly focused on cardiovascular disorders, including coronary heart disease and heart failure. The major group studied consisted predominantly of individuals with chronic heart failure. Some of these articles used remote monitoring technology, such as remote heart rate monitors at home, to guide patients to perform cardiac rehabilitation (CR) and exercise. The main indicators that researchers focused on through remote technology included peak aerobic capacity, peak oxygen uptake (VO2), medium and long-term hospitalization rate of the patient, cardiac mortality, and all-cause mortality (17). The accelerometer was used to measure the daily step count. The accelerometer was used to measure the daily step count. Moreover, the primary questionnaires provided were the International Physical Activity Questionnaire, the Health-Related Quality of Life questionnaire (evaluated using the HeartQol questionnaire), and the SF-36 Quality of Life Questionnaire. Remote monitoring methods involved mobile heart rate monitoring devices combined with SMS and email, semi-automated teaching or online video conferencing, and a smartphone-based remote home CR platform.

The VOSview and CiteSpace software were used to examine the progression patterns in the field. A bibliometric analysis was conducted on the main authors in the research field, the national institutions with significant output and close collaboration, the primary journals, the important references, and the most frequently searched and used keywords in the field. The 10 most cited articles on the cardiovascular system were identified, and seven of the articles/studies/guidelines were related to the rehabilitation of patients with CVDs and the key indicators of rehabilitation.

The low participation rate of traditional CR can be attributed to issues such as cost-effectiveness (18). However, the advancement of teletechnology has led to the development of home-based telecardiac rehabilitation, which has improved the potential for participation. TeleCR refers to the complete remote care of cardiac diseases by monitoring vital signs, providing exercise training, offering dietary guidance, educating on smoking cessation and disease, and providing psychological therapy (19). The two main types of remote devices for remote CR are a dynamometer and a wearable watch (20, 21). In a study by Maddison R., a smartphone app and wearable sensors were utilized to gather data on the patient's heart rate, respiratory rate, and other factors. The study revealed that remote CR had comparable results regarding the patient's VO2, waist circumference, and hip circumference. Additionally, remote CR was more cost-effective and lowered the drug cost (19).

Kraal (22) and Maddison (19) studied and compared the efficacy and cost-effectiveness of telecardiac rehabilitation vs. traditional CR. The study showed that the effect of home-based telecardiac rehabilitation did not differ much from that of the conventional CR and was more cost-effective. In a study published in 2021, Kikuchi (23) examined home telerehabilitation in older adult patients with heart failure and observed better patient compliance and a longer distance in the 6 min walking experiment. The 6 min walk and cardiopulmonary exercise tests can determine the patient's maximum exercise capacity, and its indicators play an important role in the CR of the patient. The study conducted by Malhotra R. examined the correlation between cardiopulmonary exercise testing and VO2 measurement, the capacity to engage in everyday activities, and the prediction of heart failure (24).

In the top 10 cited articles related to the cardiovascular system, the study by Dimeo F. performed 24 h ambulatory blood pressure monitoring and found that physical activity reduced blood pressure in patients with resistant hypertension; the guidelines also included suggestions for ambulatory monitoring in people with hypertension (22, 25). Gardner A. W. conducted a controlled trial to compare home exercising and supervised care to usual treatment in patients with intermittent claudication and peripheral artery disease. They used a step activity monitor to measure the outcomes. The study found that patients who participated in home-based exercise had higher adherence rates and experienced better claudication symptoms (26). Another study published in 2014 reported that home exercise for patients with peripheral vascular disease using teletechnology was associated with less staff supervision attrition, higher patient compliance, and overall cost-effectiveness (27).

The study by Nakayama (28) also showed that, to date, no significant risk has been reported in the trials of remote CR. Attention should be directed towards the suitable groups for remote CR, including patients residing in geographic locations and those without cognitive impairment. Patients with difficulty using electronic devices, such as older adult patients, find it challenging to perform telerehabilitation, and those with a high risk of exercise must also be carefully considered when performing telerehabilitation.

After a detailed review, it was found that the application of remote technology in rehabilitating patients with heart and large blood vessel disease mainly focuses on community and family telerehabilitation. However, few studies have been conducted on patients' rehabilitation while in the hospital, and the researchers think this might be because bedside monitoring of patients has become the standard. The rehabilitation of patients with heart and large blood vessel disease is a long-term process, and the rate of rehabilitation implementation of cardiac patients after discharge is not high, with the implementation being difficult; hence, more in-depth research is warranted. Through an analysis of the current research on the use of remote technology in rehabilitating patients with heart and large blood vessel disease, the aim was to offer innovative approaches for the rehabilitation of patients with these conditions and provide guidance for clinical practitioners in this field.

#### 3.2.2 Research hotspots and future trends

A term and subject keyword burst analysis was conducted of keywords and the literature included in the past five years (2019–2023), Figures 11, 12. After 2020, cardiac rehabilitation, remote monitoring, and secondary prevention emerged as new research hotspots. Researchers speculated that this might be because of the new crown pneumonia epidemic, which sparked the epidemic of cardiac rehabilitation and remote monitoring. Other popular research fields in the last five years include engineering and biomedicine, telecommunications, computer science, information systems, psychology, clinical, veterinary science, nuclear medicine, imaging medicine, clinical neurology, and nutrition.

**Figure 11:**
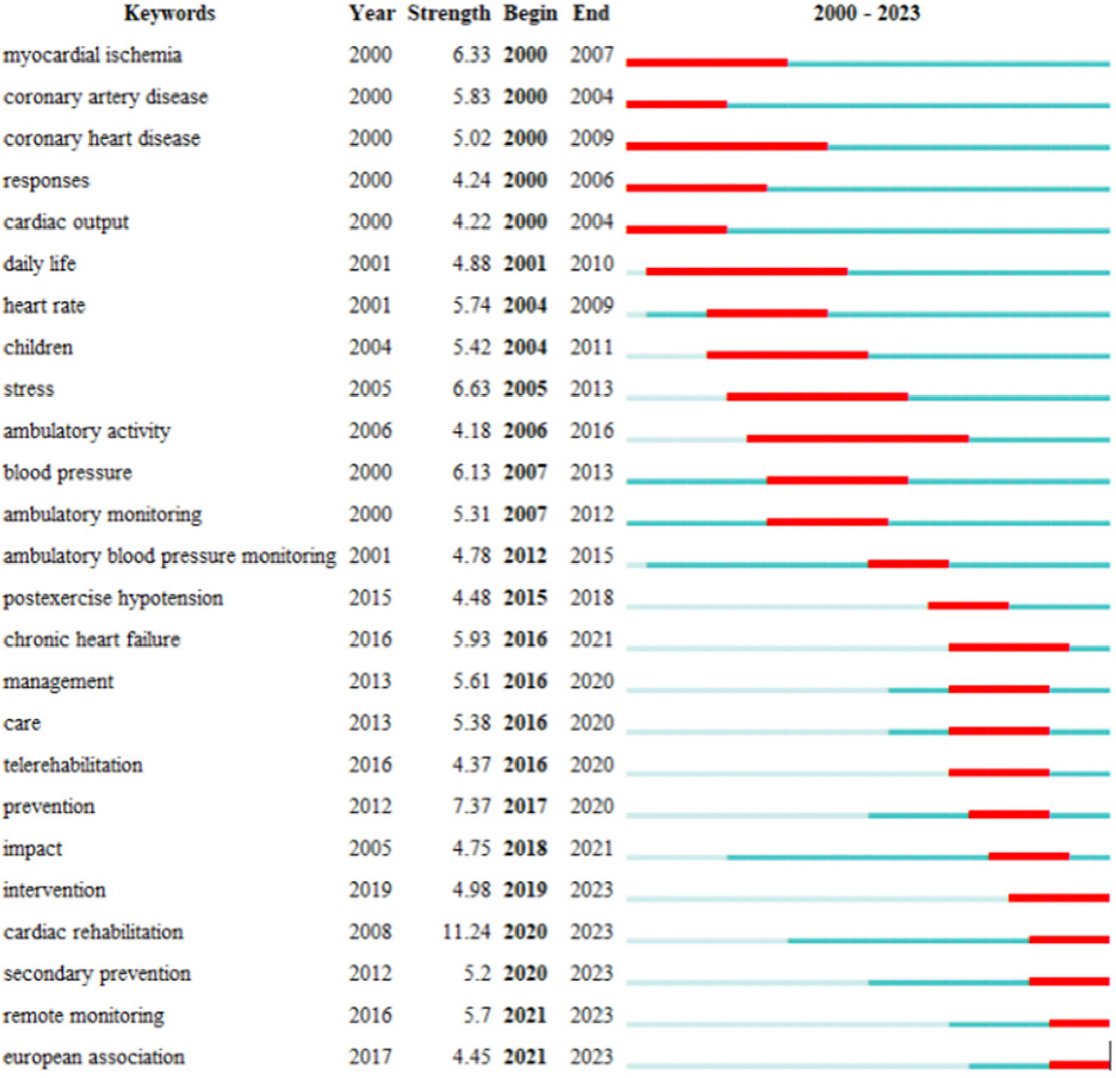
Top 25 keywords with the strongest citation brsts.

**Figure 12:**
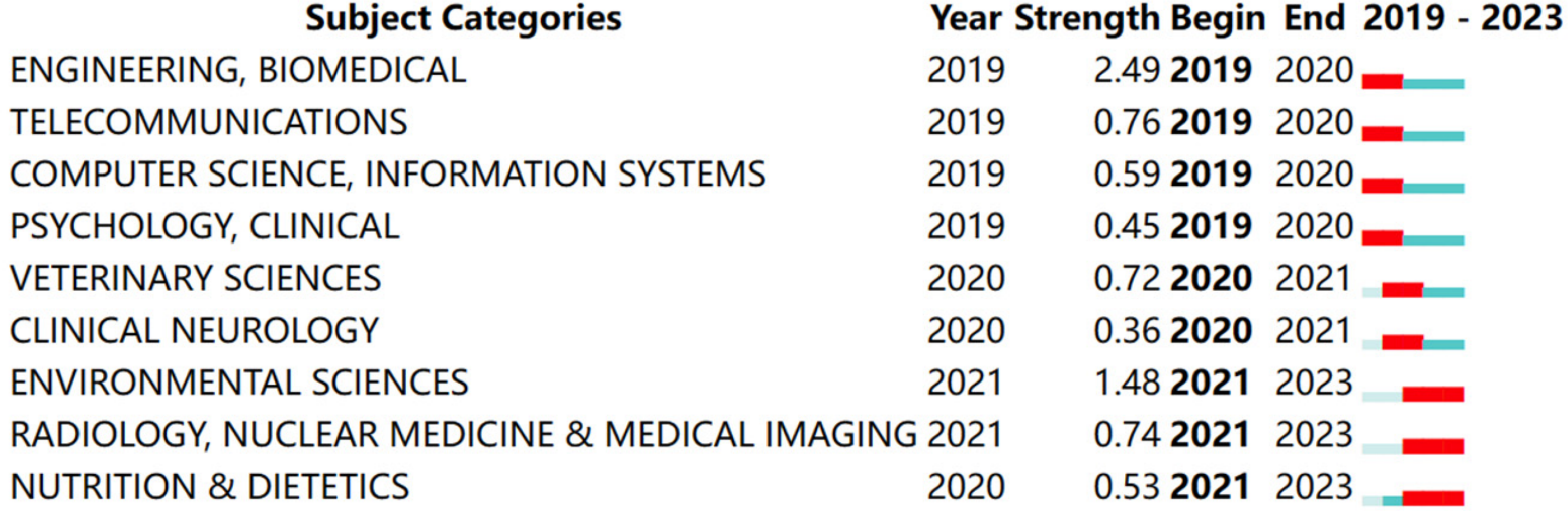
Top 9 subject categories with the strongest citation bursts.

### 3.3 Study limitations

Like most similar studies, this study has some limitations. One of its primary limitations is that the study is limited to papers indexed in the WOS core database that date back to the 21st century. Furthermore, the study has specific qualitative components, which might introduce some bias in selecting and labeling the research topic. At the same time, we did not impose age restrictions on the enrolled patients, which may result in a diverse study population. Future analyses could focus on specific subgroups, such as individuals over 80 years of age, who have the highest prevalence of CVD.

## 4 Conclusion

Research on the use of teletechnology in patients with heart and large blood vessel diseases is a multidisciplinary field that combines clinical and rehabilitation medicine. Teletechnology has garnered significant interest from several sectors and is a subject of ongoing and evolving study. The present investigation examined the current state and potential future research directions of teletechnology in patients with this condition based on research conducted in the 21st century. The review revealed Kim S., Williams L. S., Anderson C., and Ali M. as important authors in this field; accordingly, among countries, the United States, the United Kingdom, and Italy have contributed the most in this field. The Harvard University and APHP were identified as significant research facilities, while CIRCULATION was recognized as the most prominent primary journal in the area. The researchers primarily focused on objective markers such as heart rate, blood pressure, and cardiac output. The researchers primarily focused on daily living, physical activity, exercise endurance, and quality of life as the key outcomes. Heart failure and coronary artery disease were the most widely studied diseases. Secondary prevention, cardiac rehabilitation, and home-based remote rehabilitation were research hotspots in recent years. This analysis will enhance academic collaboration and exchanges among countries, institutions, and scholars. It will also serve as a valuable resource for researchers seeking to gain a deeper understanding of the current research areas, priorities, and emerging trends in the field of teletechnology and rehabilitation for patients with heart and large blood vessel diseases.
